# Correction: Cu(ii)/Ni(ii)–organic frameworks constructed from the homometallic clusters by 5-(2-carboxyphenoxy)isophthalic acid and N-ligand: synthesis, structures and visible light-driven photocatalytic properties

**DOI:** 10.1039/c9ra90043h

**Published:** 2019-06-03

**Authors:** Qi-Wei Xu, Qiu-Shuang Wang, Shan-Shan Li, Xia Li

**Affiliations:** Department of Chemistry, Capital Normal University Beijing 100048 China xiali@cnu.edu.cn +86 1068902320 +86 1068902320

## Abstract

Correction for ‘Cu(ii)/Ni(ii)–organic frameworks constructed from the homometallic clusters by 5-(2-carboxyphenoxy)isophthalic acid and N-ligand: synthesis, structures and visible light-driven photocatalytic properties’ by Qi-Wei Xu *et al.*, *RSC Adv.*, 2019, **9**, 16305–16312.

In the Acknowledgements section, a funder, Laboratory Open Project Fund of Capital Normal University (LIP18009S047) was omitted.

The revised acknowledgements should read:

The authors are grateful to the Key Project of Science and Technology plan of Beijing Education Commission (KZ201910028038), the National Natural Science Foundation of China (21471104) and Laboratory Open Project Fund of Capital Normal University (LIP18009S047).

The Royal Society of Chemistry apologises for these errors and any consequent inconvenience to authors and readers.

## Supplementary Material

